# Exploring the link between environmental pollution and economic growth in EU-28 countries: Is there an environmental Kuznets curve?

**DOI:** 10.1371/journal.pone.0195708

**Published:** 2018-05-09

**Authors:** Daniel Armeanu, Georgeta Vintilă, Jean Vasile Andrei, Ştefan Cristian Gherghina, Mihaela Cristina Drăgoi, Cristian Teodor

**Affiliations:** 1 Department of Finance, The Bucharest University of Economic Studies, Bucharest, Romania; 2 Business Administration Department, Petroleum-Gas University of Ploiesti, Ploiesti, Prahova, Romania; 3 Department of International Business and Economics, The Bucharest University of Economic Studies, Bucharest, Romania; 4 Department of Agri-Food and Environmental Economics, The Bucharest University of Economic Studies, Bucharest, Romania; Universitat Jaume I, SPAIN

## Abstract

This study examines the Environmental Kuznets Curve hypothesis (EKC), considering the primary energy consumption among other country-specific variables, for a panel of the EU-28 countries during the period 1990–2014. By estimating pooled OLS regressions with Driscoll-Kraay standard errors in order to account for cross-sectional dependence, the results confirm the EKC hypothesis in the case of emissions of sulfur oxides and emissions of non-methane volatile organic compounds. In addition to pooled estimations, the output of fixed-effects regressions with Driscoll-Kraay standard errors support the EKC hypothesis for greenhouse gas emissions, greenhouse gas emissions intensity of energy consumption, emissions of nitrogen oxides, emissions of non-methane volatile organic compounds and emissions of ammonia. Additionally, the empirical findings from panel vector error correction model reveal a short-run unidirectional causality from GDP per capita growth to greenhouse gas emissions, as well as a bidirectional causal link between primary energy consumption and greenhouse gas emissions. Furthermore, since there occurred no causal link between economic growth and primary energy consumption, the neo-classical view was confirmed, namely the neutrality hypothesis.

## Introduction

Energy is a crucial resource for any economy since all production and consumption undertakings are directly connected to energy consumption, hence ensuring the basis for economic activity and social welfare; however, the use of fossil fuels as primary source of energy caused a noteworthy upsurge in the global emissions of several potentially harmful gases [1]. Greenhouse gases (GHGs) absorb heat arriving from the Sun and retain it in the atmosphere which entails the increase of earth surface temperature [2]. Therefore, the matter of climate change driven by the increased quantity of GHGs polluting the atmosphere has depicted over the past two decades a major environmental concern caused by the phenomenon of global warming. However, the switch between renewable resources and fossil fuels is at the core of climate change mitigation strategy [3].

The CO_2_ emissions generated by the gaseous/liquid/solid fuel consumption are liable for more than 60% of the greenhouse effect [4] and represent the leading power source of the industry in general and of the automobile industry in particular, which are directly associated with economic growth and development [5]. While [6] forecasted a mean annual growth of emissions of approximately 1.8% until 2025, the most recent report of International Energy Outlook [7] estimated an increase of 34% of world energy-related CO_2_ emissions in 2040 relative to 2012, the developing non-OECD countries being held accountable for a considerable share, since they remain reliant on fossil fuels to fulfill the upsurge in energy demand. However, there is an anticipated depletion of oil resources, natural gas and uranium within a few decades and coal in almost two centuries [8]. Consequently, the United Nations Environment Programme via the International Resource Panel proposed a strategic approach towards a low carbon resource efficient Green Economy that pursues decoupling human wellbeing from resource consumption [9]. Thereby, relative decoupling supposes a slight rate of increase in resource employment compared to the growth rate of the economy, whilst absolute decoupling reveals a whole decline of resource use as an economy grows [10]. Alike, the European Union (EU) Sustainable Development Strategy [11] seeks to hinder and reduce environmental pollution, therefore supporting sustainable consumption and production in order to break the connection between economic growth and ecological deprivation. Consistent with the second commitment phase of the Kyoto Protocol 2013–2020 [12], EU set a 20% reduction target of GHG emissions to be achieved by 2020 compared to the 1990s level [13], and also a decrease by at least 40% below 1990 levels by 2030 [14].

Previous Environmental Kuznets Curve (EKC) studies on Europe explored various countries such as Austria [15], Croatia [16], France [17], Italy [18, 19], Portugal and Spain [20], Romania [21], Spain [22–25], United Kingdom [26]. However, to the best of our knowledge, there are not many studies that have explored the EKC hypothesis for pollutant emissions across EU-28. For instance, [27] tested the EKC hypothesis for municipal solid waste generation for a data panel consisting of 32 European states over 1997–2010. [28] explored the causal link between energy consumption, carbon dioxide emissions, economic growth, trade openness and urbanization, but for a panel of new EU member and candidate countries over the period 1992–2010. [29] studied the causal association between economic growth and CO2 emissions in a panel of 24 European nations from 1980 to 2010. Even if [30] selected EU-28 countries, the study analyzed the connections between residential energy consumption and income, within the timeframe between 1990–2013. Also, [31] investigated the transport energy by using Environmental Kuznets Curve hypothesis in the EU-27 countries over 1995-2009. The main features that distinguish the current paper from previous research on the topic are the research sample, as well as the employment of several types of emissions apart from carbon dioxide (CO_2_), namely sulfur oxides (SOx), nitrogen oxides (NOx), non-methane volatile organic compounds (NMVOCs), ammonia (NH_3_). The focus on EU-28 states is justified in the context of 2030 Framework for climate and energy [32] which put forward targets for greenhouse gas emissions decrease and renewable energy as part of the EU move to a competitive low carbon economy. Following a classical, logical structure, the next section highlights the literature review related to the EKC hypothesis, as well as to the relationship between CO_2_ emissions and economic growth. The data used for analysis, alongside the econometric framework are presented in Section 3. The empirical results and discussions are shown in Section 4, while the final section concludes the paper and provides policy implications.

## Literature review

### Previous evidence on Environmental Kuznets Curve

An extensive part of the specific literature explores the association between national income levels and the demand for greater environmental quality, namely the EKC hypothesis. According to [33], income inequity first rises with economic progress and then drops as economy advances to a certain level. Following the same reasoning, the EKC hypothesis points out that intensification in economic growth will primarily cause environmental pressure, but after a particular stage, increase in economic growth will lessen the environmental pressure. Specifically, EKC expects an inverted U-shaped association between environmental degradation and economic growth. Per se, initially economic growth will amplify CO_2_ emanations, but after a certain level (turning point) this connection will come to be the opposite. For that reason, after a certain period, upsurge in economic growth will lessen CO_2_ emissions, accordingly, economic growth itself being the way out for an uncontaminated setting. In Table 1 are summarized the turning points identified in earlier studies, by type of pollutant.

**Table 1:** Turning points reached in previous studies, by type of pollutant.

Pollutant type	Study	Dataset	Period	Econometric technique	Turning point
Greenhouse gas emissions	[6]	108 states	1951–1986	Fixed effects regressions	35,428 (in 1986 U.S. $)
Greenhouse gas emissions	[34]	OECD and non-OECD states	1960–1998	Weibull specification	15,599.90–21,185.83 (in 1990 U.S. $)
Greenhouse gas emissions	[35]	16 metropolitan regions in Korea	1990–2005	Fixed effects and random effects regressions	26,400–30,000 (in 2000 U.S. $)
Greenhouse gas emissions	[36]	12 Middle East and North African states (MENA)	1981–2005	Panel Error-Correction	37,263 (in 2005 international $)
Greenhouse gas emissions	[25]	Spain	1874–2011	Autoregressive distributed lag (ARDL)	8,103 (in 1990 U.S. $)
Greenhouse gas emissions	[37]	136 states	1971–2010	Ordinary least squares (OLS) with heteroskedasticity-robust standard errors	outside the sample and statistically insignificant
Greenhouse gas emissions	[38]	China	1970–2015	Auto regressive distributed lag (ARDL), fully modified ordinary least squares (FMOLS), dynamic ordinary least squares (DOLS) and impulse response and variance decomposition	744,665 (in 2010 U.S. $)
Greenhouse gas emissions	[39]	Morocco	1966–2014	Ordinary least squares (OLS)	7,800 (in 2010 U.S. $)
Emissions of sulfur oxides	[40]	Cross-section of urban areas located in 42 countries	1977; 1982; 1988	Random effects regressions	4,000–5,000 (in 1985 U.S. $)
Emissions of sulfur oxides	[41]	149 states	1960–1990	Ordinary least squares (OLS)	3,670 (in 1985 U.S. $)
Emissions of sulfur oxides	[42]	55 states	1987–1988	Ordinary least squares (OLS)	2,900–3,800 (in 1985 U.S. $)
Emissions of sulfur oxides	[43]	30 states	1979–1987	Fixed effects regressions	8,700–10,700 (in 1990 U.S. $)
Emissions of sulfur oxides	[44]	58 Turkish provinces	1992–2001	Pooled EGLS (cross-section weights)	1,934 and 5,817 (in 2000 U.S. $)
Emissions of sulfur oxides	[45]	Tunisia	1961–2004	Johansen cointegration	1,200 (in 2000 U.S. $)
Emissions of sulfur oxides	[35]	16 metropolitan regions in Korea	1990–2005	Fixed Effects and Random Effects Regressions	5,700 and 28,000 (in 2000 U.S. $)
Emissions of sulfur oxides	[37]	136 states	1971–2010	Ordinary least squares (OLS)	112,000 (in 2005 PPP adjusted U.S. $)
Emissions of nitrogen oxides	[42]	55 states	1987–1988	Ordinary least squares	5,500 (in 1985 U.S. $)
Emissions of nitrogen oxides	[35]	16 metropolitan regions in Korea	1990–2005	Fixed Effects and Random Effects Regressions	27,600 (in 2000 U.S. $)

Source: Authors’ compilation based on literature review.

The starting point of the EKC pertains to [40] which showed that there is an inverted U-shaped relation between income level and environmental pressure; their findings were continued by [41–43]. Nevertheless, the EKC empirical evidence is still questioned and there is no consensus on the income level at which environmental degradation starts diminishing [46]. However, [47] noticed that the EKC is an important empirical factor, but most of the EKC studies are econometrically weak.

Likewise, [48] argued that the empirical outcomes highlight the omission of explanatory variables, while [49] noticed the use of dissimilar environmental quality indicators, estimation methods, economic features, and period covered. According to [50, 51], three approaches were observed at aiming to shed light on the inverted U-shaped link between pollutants and output.. *The scale effect* presumes that emissions are likely to increase as the number and variety of economic activity rise. *The composition effect* assumes that emissions would decrease as long as the goods produced in an economy become cleaner. *The technique effect* considers that emissions would decrease as the knowledge implied in manufacturing becomes less polluting. Besides, [52] explored the contribution of education by considering carbon dioxide emissions in Australia over 1950–2014 and find that increase in education rate has gradually counterbalanced the growth of per capita CO2 emissions ensuing from the economic growth.

The central assumption of EKC, respectively that global income is normally distributed and that all nations are supposed to follow a common development pattern is also suspicious [53]. Accordingly, inconsistent conclusions towards EKC come from cross-country examinations. In this regard, for two different datasets comprising OECD and non-OECD states, [34] provided evidence for EKC only for the OECD countries by estimating several linear or log-linear regression equations among variables, as well as quadratic or cubic. [54] supported the EKC for 43 developing countries over 1980–2004 via panel co-integration and panel long-run estimation techniques.

[55] employed the Johansen co-integration test and found evidence for the EKC in the case of low and lower middle income countries over 1975–2014, but failed to support the validity of EKC in the case of upper middle income and high income countries. [56] showed by using fully modified ordinary least squares (FMOLS) and dynamic ordinary least squares (DOLS) that the EKC is valid for a panel of 25 OECD states during 1980–2010. [57] supported the EKC in the energy-resource depletion model, for a panel of nine developed countries covering the period 2000–2013 through panel generalized method of moments (GMM). [36] applied panel unit root tests and co-integration techniques and did not confirm the EKC for 12 Middle East and North African Countries (MENA) over 1981–2005, except for Jordan. [58] used co-integration and Granger causality methods and revealed that EKC is not valid for ASEAN-5 economies, particularly for Indonesia, Malaysia, and Thailand. The results of the FMOLS panel estimator employed by [59] for 12 Middle East countries over 1990–2008 yielded evidence divergent to the EKC.

[60] estimated pooled, fixed effects, random effects, and generalized-least square regressions, but did not validate the EKC for 152 states over six years. Furthermore, contradictory evidence towards EKC hypothesis is also established in single country studies. [61] found that nuclear energy has a positive influence on environmental quality in Korea over 1971–2007 by employing the autoregressive distributed lag (ARDL) approach to co-integration. By using threshold co-integration tests, [62] revealed that EKC hypothesis is valid in India’s case during 1971–2008. [63] underlined the validity of EKC in Turkey over 1961–2010 using the ARDL method.

[64] used the Spatial Durbin Model and supported the presence of the EKC for per capita coal consumption in China between 1995 and 2012. [65] validated the EKC in Indonesia for the period 1971–2010 by utilizing ARDL method. In the case of Qatar, [66] employed the ARDL method over the timeframe 1980–2011 and rejected the EKC hypothesis when using carbon dioxide emissions, but confirmed the EKC when using ecological footprint. Otherwise, the pooled mean group estimates undertaken by [67] reject the EKC for the case of US over 1945–2004. As well, apart from the inverted U-shaped relationship between environmental degradation and economic growth, other findings pointed out diverse shapes. As such, monotonically decreasing curve denotes that environmental quality improves as income growths, whilst monotonically increasing curve involves the diminution of environmental quality as income rises. Moreover, the N-shaped curve unveils that environmental degradation appears again after a decline to a specific level.

Furthermore, by applying panel unit root and panel co-integration tests, [68] rejected the EKC for a sample of 21 Latin American and Caribbean nations during 1970–2007. By employing a multivariate vector error correction model (VECM), [69] did not confirm also the EKC hypothesis in Russia between 1990 and 2007 and neither did [70] for Cambodia over 1996–2012, by estimating GMM and two-stage least square regression models. [71] used the STIRPAT empirical model, as well as panel co-integration and FMOLS, but failed to support the EKC in five African states during 1980–2011.

A brief literature review that found different patterns on this topic is described in Table 2. However, even if [72] confirmed the EKC hypothesis, there was argued that the results cannot be generalized, being fragile without performing sensitivity analysis.

**Table 2:** Summary review of literature invalidating EKC hypothesis.

Study	Dataset	Period	Econometric technique	Outcome
[73]	Chinese provincial level	1985–2015	VECM	EKC at aggregate-level for SO_2_ EKC may not exist at the provincial-level for SO_2_
[74]	100 states	1960–1996	Nonparametric panel model with individual effects	Upward sloping curve for CO_2_
[44]	Turkey	1968–2003 1992–2001	Johansen technique, Feasible Generalized Least Squares	Monotonically increasing curve for CO_2_ N-shaped for SO_2_ and PM_10_
[45]	Tunisia	1961–2004	Johansen technique, Granger causality	Monotonically increasing curve for CO_2_
[35]	Korea	1990–2005	Fixed-effects, Random-effects, Random coefficient regressions	Potential N-shaped curve for SO_2_ Dominant U-shaped curve for CO A region-specific U-shaped curve for NO_2_
[75]	8 states	1970–2010	ARDL	Inverted U-shaped curve for CO_2_ in Japan and South Korea N-shaped curve for Brazil, China, Egypt, Mexico, Nigeria, and South Africa
[76]	25 Sub-Saharan Africa states	1996–2010	Ordinary Least Squares, Difference GMM, System GMM, Least Square Dummy Variable Corrector	Monotonically increasing curve for CO_2_
[77]	189 states	1990–2012	Fixed-effects and Random-effects panel regressions, Dynamic panel regressions, Heterogeneous panel regressions	Linearly increasing curve for CO_2_
[78]	China	1997–2012	Non-spatial panel models and spatial Durbin model	Inverted N-shaped curve for CO_2_
[51]	17 OECD states	1990–2012	Fixed-effect panel regressions	N-shaped curve for per capita GHG emissions
[79]	India, China	1971–2012	ARDL	N-shaped curve for CO_2_
[80]	Saudi Arabia	1970–2014	ARDL	GDP growth and CO_2_ emissions are positively and linearly associated

Source: Authors’ compilation based on literature review.

### Previous evidence on the causal link between CO2 emissions and growth

Other part of literature examines the causal relationship between energy consumption and economic growth. The pioneering examinations with regard to the economic growth—energy consumption connection belongs to [81] which found an unidirectional causality between the gross national product and energy consumption for the United States, whilst [82] did not provide any causal link.

There are two prevailing divergent hypothetical points of view on the connection between energy consumption and economic growth [83, 84]. The neo-classical view, known as the neutrality hypothesis [85, 86], claims that there are other significant factor inputs in the production process than energy and no causal link occurs between energy consumption and economic growth. Thus, a nation may follow a protectionist energy strategy for lowering CO_2_ emanations deprived of compromising growth. The antagonist view, referred as the non-neutrality hypothesis of energy, suggests that energy is a fundamental factor input in the production process and energy conservation policies may obstruct the economic growth. Subsequently, three theoretical models derive from the latter approach [87]. *The growth hypothesis* [88–92] advocates a uni-directional causality from energy consumption to economic growth and pretends that energy conservation policies will have negative effects on economic growth. *The conservation hypothesis* [93, 94] supports a uni-directional causality from economic growth to energy consumption and motivates that energy conservation policies will not impair economic growth.

*The feedback hypothesis* [104–107] postulates a bidirectional causality between energy consumption and economic growth and asserts that energy conservation policies may weaken economic growth performance, so growth variations are reflected back to energy consumption. Overview of the studies that investigated the causal link between CO_2_ emissions and economic growth is showed in Table 3.

**Table 3:** Brief literature review on the causal relation between CO_2_ emissions and economic growth.

Study	Dataset	Period	Econometric technique	Outcome
[95]	88 states	1960–1990	Co-integration analysis, Error Correction Model	Bidirectional causality between per capita CO2 emissions and per capita GDP for country-group of Africa Unidirectional causality running from per capita GDP to per capita CO2 emissions for country-group of Central America Unidirectional causality running from per capita CO2 emissions to per capita GDP for country-group of Europe
[4]	Turkey	1968–2005	ARDL	Lack of causality between per capita CO2 emissions and real GDP per capita
[96]	China, India	1965–2009	Co-integration analysis, Granger causality analysis	Unidirectional causality running from economic growth to CO2 emissions
[97]	Malaysia	1980–2009	Johansen-Julius co-integration, ARDL, VECM	Bidirectional causality between economic growth and CO2 emissions
[98]	Indonesia	1975–2011	ARDL, VECM	Bidirectional causality between economic growth and CO2 emissions
[99]	BRICS states	1990–2010	Panel causality analysis	CO2–GDP feedback for Russia Unidirectional causality running from GDP to CO2 in South Africa Unidirectional causality running from CO2 to GDP in Brazil
[100]	54 states	1990–2011	Dynamic simultaneous-equation panel data models	Unidirectional causality running from CO2 emissions to economic growth for Europe and Central Asia, Latin America and the Caribbean Bidirectional causality between CO2 emissions and economic growth for Middle Eastern, North African, and sub-Saharan panel
[101]	51 states	1995–2013	Simultaneous-equation models estimated by the GMM.	Bidirectional causal relationships between CO2 emissions and economic growth
[102]	17 MENA states	1990–2012	Simultaneous-equation panel data VAR model	Unidirectional causality running from economic growth to CO2 emissions
[84]	Pakistan	1971–2009	Johansen-Julius co-integration, ARDL, VECM	Bidirectional causalities between energy consumption, economic growth and the CO2 emissions
[103]	G7 states	1820–2015	Nonparametric co-integration, Causality tests, Cross-validated local linear regression	Nonlinear causal relationship between CO2 and economic growth

Source: Authors’ compilation based on literature review.

## Data and methodology

### Sample and variables

Our data sample covers the period 1990–2014 for a panel consisting of the EU-28 countries. The variables used for analysis, as well as their definition and data sources are presented in Table 4. All variables, except GDPCG, ENVTR, GFCF, RD, FFEC, EMPL, INDVA, and CPI are expressed in natural logs.

**Table 4:** Description of the variables used for analysis.

Variables	Definition	Unit of measurement	Time frame availability	Data source
GDPC	GDP per capita	Constant 2010 US dollars	1990–2015	World Bank (NY.GDP.PCAP.KD)
GDPCG	GDP per capita growth	Annual %	1990–2015	World Bank (NY.GDP.PCAP.KD.ZG)
GGE	Greenhouse gas emissions	Tons per capita	1990–2014	Eurostat (sdg_13_10)
GGEI	Greenhouse gas emissions intensity of energy consumption	Index (2000 = 100)	1990–2014	Eurostat (tsdcc220)
ESOX	Emissions of sulfur oxides	Tons	1990–2014	Eurostat (tsdpc260)
ENOX	Emissions of nitrogen oxides	Tons	1990–2014	Eurostat (tsdpc270)
ENMVOC	Emissions of non-methane volatile organic compounds	Tons	1990–2014	Eurostat (tsdpc280)
ENH3	Emissions of ammonia	Tons	1990–2014	Eurostat (tsdpc290)
GGET	Greenhouse gas emissions from transport	Million tons of CO2 equivalent	1990–2014	Eurostat (tsdtr410)
ENVTR	Environmental tax revenues	% of total revenues from taxes and social contributions	1995–2015	Eurostat (t2020_rt320)
PEC	Primary energy consumption	Million tons of oil equivalent (TOE)	1990–2015	Eurostat (tsdcc120)
GIECRE	Gross inland consumption of renewable energies	1,000 tons of oil equivalent	1990–2015	Eurostat (tsdcc320)
GFCF	Gross fixed capital formation	% of GDP	1995–2016	Eurostat (tipsna20)
RD	Research and development expenditure	% of GDP	1996–2014	World Bank (GB.XPD.RSDV.GD.ZS)
FFEC	Fossil fuel energy consumption	% of total	1990–2015	World Bank (EG.USE.COMM.FO.ZS)
EMPL	Employment in high- and medium-high-technology manufacturing sectors	% of total employment	1995–2014	Database for Institutional Comparisons in Europe (DICE)
INDVA	Industry, value added	% of GDP	1990–2016	World Bank (NV.IND.TOTL.ZS)
CPI	Corruption Perceptions Index	Score	1995–2016	Database for Institutional Comparisons in Europe (DICE)

Source: Authors’ own selection based on databases’ availability.

A part of preceding studies that tested the EKC hypothesis used only pollutant emissions, economic growth, and energy consumption [2, 21, 24, 28, 36, 59, 60, 104], while other examinations furthermore considered new variables such as electricity consumption, trade openness, corruption, and government effectiveness index [70], electricity production [61], oil prices [25], population and urbanization [44, 55, 71, 78], trade [1, 56, 58, 62, 66, 75, 79, 80]. In line with [108–110] we control for the technology level via FFEC and EMPL. We employ the fossil fuel energy consumption to account for the level of use of dirty energy, which causes air pollution in the combustion process. As well, the employment in high- and medium-high-technology manufacturing sectors is included in as much as highly skilled labor contributes to the development of an efficient fabrication process that can stimulate energy savings and pollution deterrence. According to [52], an advanced level of comprehension to integrate cleaner technologies and collective responsiveness among people will drive higher ecological standards. INDVA is applied to control for the scale, whilst CPI aims to control for the selected states political state. Corrupted or unstable political regimes damage industrial progress and destroy the application of environmental policy in pollution abatement undertakings [110]. In the same vein, [111] concluded that prior communist nations had trouble introducing modern productive equipment conceived in capitalistic countries and depended on old and inefficient tools. On the contrary, [112] revealed that regulatory stringency positively influence efficiency, arguing that more rigorous emission standards lead firms to search for more efficient methods of fuel use and emissions decrease. However, further country-specific variables employed within current research are environmental tax revenues, gross inland consumption of renewable energies [51, 56], as well as gross fixed capital formation and research and development expenditure [51, 65, 85]. According to [113] amongst nations variation systematically differs conditional on its richness, on institutional features, and on its whole commitment to the diffusion of renewable energy.

### Econometric methods

In order to examine the EKC hypothesis we followed the approach of [35, 44, 51, 76, 77, 104]. The long-run relationship between pollutant emissions, GDP per capita, environmental tax revenues, primary energy consumption, gross inland consumption of renewable energies, gross fixed capital formation, research and development expenditure, and other cross-country control variables, is given as follows:

$$\begin{array}{l} {\text{PE}}_{\text{it}}=\alpha_{\text{it}}+\delta_{1\text{i}}{\text{GDPC}}_{\text{it}}+\delta_{2\text{i}}{\text{GDPC}}_{\text{it}}^{2}+\delta_{3\text{i}}{\text{ENVTR}}_{\text{it}}+\delta_{4\text{i}}{\text{PEC}}_{\text{it}}+\delta_{5\text{i}}{\text{GIECRE}}_{\text{it}}+\delta_{6\text{i}}{\text{GFCF}}_{\text{it}}+\delta_{7\text{i}}{\text{RD}}_{\text{it}}+\delta_{8\text{i}}{\text{FFEC}}_{\text{it}}+\\ \delta_{9\text{i}}{\text{EMPL}}_{\text{it}}+\delta_{10\text{i}}{\text{INDVA}}_{\text{it}}+\delta_{11\text{i}}{\text{CPI}}_{\text{it}}+\varepsilon_{\text{it}} \end{array}$$ (1)

where *i = 1*, …, *28* and *t = 1990*, …, *2014* reveal the country and time, respectively, whereas *PE* denotes the pollutant emissions which take form of greenhouse gas emissions, greenhouse gas emissions intensity of energy consumption, emissions of Sulphur oxides, emissions of nitrogen oxides, emissions of non-methane volatile organic compounds, emissions of ammonia, and greenhouse gas emissions from transport. α_it_ indicates the country specific fixed effect.

The parameters δ_1i_–δ_11i_ are the long-run elasticities related to each explanatory variable of the panel. ε_it_ describes the estimated residuals which characterize deviations from the long-run equilibrium.

Considering the inverted U-shaped EKC hypothesis, the sign of δ_1i_ is expected to be positive and the sign of δ_2i_ is expected to be negative, whilst the monetary value representing the turning point is computed by τ = exp[-*β*_1_/(2*β*_2_)] [23, 49, 60, 76].

Furthermore, our aim was to establish the causal links between greenhouse gas emissions, economic growth, primary energy consumption, and environmental tax revenues.

Therefore, according to [59, 84, 88, 89, 104], we assessed the stationarity of data using a battery of first generation tests, such as Levin, Lin and Chu (LLC), Im, Pesaran and Shin (IPS), Augmented Dickey-Fuller (ADF), and Phillips-Perron (PP), as well as second generation checks, namely the cross-sectionally augmented Dickey-Fuller (CADF). However, the IPS unit root test [114] allows for heterogeneous autoregressive coefficients.

Subsequently, we performed the heterogeneous panel co-integration test proposed by Pedroni [115, 116] since it allows cross-section interdependence with different individual effects, alongside Kao [117] and Johansen approaches [118], as well as Westerlund [119]:

$${\text{GDPCG}}_{\text{it}}=\alpha_{\text{i}}+\delta_{\text{i}}{\text{t}+\gamma }_{1\text{i}}{\text{GGE}}_{\text{it}}+\gamma_{2\text{i}}{\text{ENVTR}}_{\text{it}}+\gamma_{3\text{i}}{\text{PEC}}_{\text{it}}+\varepsilon_{\text{it}}$$ (2)

where *i = 1*, …, *28* for each country in the panel and *t = 1990*, …, *2014* denotes each year of the period. Besides, the parameters α_i_ and δ_i_ allow country-specific fixed effects and deterministic trends. By pursuing the two-step procedure of Engle-Granger, the long-run model specified in Eq (2) is estimated in which the one period lagged residuals serve as the error correction term.

The dynamic error correction model is presented below:

$$\begin{array}{l} \Delta {\text{GDPCG}}_{\text{it}}=\alpha_{1\text{j}}+\overset{\text{q}}{\underset{\text{k}=1}{\sum }}\varphi_{11\text{ik}}\Delta {\text{GDPCG}}_{\text{it}-\text{k}}+\overset{\text{q}}{\underset{\text{k}=1}{\sum }}\varphi_{12\text{ik}}\Delta {\text{GGE}}_{\text{it}-\text{k}}+\overset{\text{q}}{\underset{\text{k}=1}{\sum }}\varphi_{13\text{ik}}\Delta {\text{ENVTR}}_{\text{it}-\text{k}}\\ +\overset{\text{q}}{\underset{\text{k}=1}{\sum }}\varphi_{14\text{ik}}\Delta {\text{PEC}}_{\text{it}-\text{k}}+\lambda_{1\text{i}}\varepsilon_{\text{it}-1}+{\text{u}}_{1\text{it}} \end{array}$$ (3a)

$$\begin{array}{l} \Delta {\text{GGE}}_{\text{it}}={\text{α}}_{2\text{j}}+\overset{\text{q}}{\underset{\text{k}=1}{\sum }}{\text{φ}}_{21\text{ik}}\Delta {\text{GDPCG}}_{\text{it}-\text{k}}+\overset{\text{q}}{\underset{\text{k}=1}{\sum }}{\text{φ}}_{22\text{ik}}\Delta {\text{GGE}}_{\text{it}-\text{k}}+\overset{\text{q}}{\underset{\text{k}=1}{\sum }}{\text{φ}}_{23\text{ik}}\Delta {\text{ENVTR}}_{\text{it}-\text{k}}+\overset{\text{q}}{\underset{\text{k}=1}{\sum }}{\text{φ}}_{24\text{ik}}\Delta {\text{PEC}}_{\text{it}-\text{k}}\\ +{\text{λ}}_{2\text{i}}{\text{ε}}_{\text{it}-1}+{\text{u}}_{2\text{it}} \end{array}$$ (3b)

$$\begin{array}{l} \Delta {\text{ENVTR}}_{\text{it}}={\text{α}}_{3\text{j}}+\overset{\text{q}}{\underset{\text{k}=1}{\sum }}{\text{φ}}_{31\text{ik}}\Delta {\text{GDPCG}}_{\text{it}-\text{k}}+\overset{\text{q}}{\underset{\text{k}=1}{\sum }}{\text{φ}}_{32\text{ik}}\Delta {\text{GGE}}_{\text{it}-\text{k}}+\overset{\text{q}}{\underset{\text{k}=1}{\sum }}{\text{φ}}_{33\text{ik}}\Delta {\text{ENVTR}}_{\text{it}-\text{k}}\\ +\overset{\text{q}}{\underset{\text{k}=1}{\sum }}{\text{φ}}_{34\text{ik}}\Delta {\text{PEC}}_{\text{it}-\text{k}}+{\text{λ}}_{3\text{i}}{\text{ε}}_{\text{it}-1}+{\text{u}}_{3\text{it}} \end{array}$$ (3c)

$$\begin{array}{l} \Delta {\text{PEC}}_{\text{it}}={\text{α}}_{4\text{j}}+\overset{\text{q}}{\underset{\text{k}=1}{\sum }}{\text{φ}}_{41\text{ik}}\Delta {\text{GDPCG}}_{\text{it}-\text{k}}+\overset{\text{q}}{\underset{\text{k}=1}{\sum }}{\text{φ}}_{42\text{ik}}\Delta {\text{GGE}}_{\text{it}-\text{k}}+\overset{\text{q}}{\underset{\text{k}=1}{\sum }}{\text{φ}}_{43\text{ik}}\Delta {\text{ENVTR}}_{\text{it}-\text{k}}\\ +\overset{\text{q}}{\underset{\text{k}=1}{\sum }}{\text{φ}}_{44\text{ik}}\Delta {\text{PEC}}_{\text{it}-\text{k}}+{\text{λ}}_{4\text{i}}{\text{ε}}_{\text{it}-1}+{\text{u}}_{4\text{it}} \end{array}$$ (3d)

where Δ signifies the first-difference operator, q represents the lag length set at one according to likelihood ratio tests, and u exposes the serially uncorrelated error term.

## Empirical results

### Summary statistics, correlations and unit root examination

Table 5 shows the descriptive statistics of the selected variables over the period 1990–2014. By type of pollutant emissions, Cyprus (GGE), Bulgaria (GGEI), Poland (ESOX), and Germany (ENOX, ENMVOC, ENH3, GGET) show the highest mean value, whereas Lithuania (GGE), Czech Republic (GGEI), Luxembourg (ESOX), and Malta (ENOX, ENMVOC, ENH3, GGET) register the lowest mean value. In terms of correlations (Table 6), strong uphill linear relationships between primary energy consumption and selected emissions such as ESOX, ENOX, ENMVOC, ENH3 and GGET were noticed.

**Table 5:** Descriptive statistics of the selected variables (raw data).

Variables	Mean	Median	Max	Min	Std. Dev.	Skewness	Kurtosis	Jarque-Bera	Prob	Obs
GDPC	28,975.18	26,046.77	111,069.20	3,582.86	19,231.61	1.33	5.89	449.0606	0.00	700
GDPCG	2.19	2.24	25.56	-14.56	3.71	-0.24	7.26	527.7636	0.00	690
GGE	11.04	10.10	35.60	4.40	4.59	1.94	9.01	1550.705	0.00	727
GGEI	100.28	100.00	138.00	77.00	8.93	0.63	4.52	113.6375	0.00	700
ESOX	396,956.60	130,796.50	5,311,611.00	16.00	625,070.50	2.93	14.48	4846.56	0.00	700
ENOX	449,170.30	205,027.50	2,949,082.00	3,834.00	588,987.30	1.87	5.81	636.4299	0.00	700
ENMVOC	387,457.70	166,212.50	3,389,448.00	1,838.00	558,010.20	2.33	8.22	1426.401	0.00	700
ENH3	150,963.10	67,826.00	792,928.00	1,498.00	190,731.40	1.78	5.26	519.789	0.00	700
GGET	32.18	12.46	186.78	0.34	45.90	1.80	4.89	483.6149	0.00	700
ENVTR	7.59	7.36	15.39	2.45	1.88	0.53	3.23	28.01873	0.00	581
PEC	57.68	24.55	333.30	0.60	78.62	1.96	5.94	727.6009	0.00	728
GIECRE	4,398.73	1,680.70	38,354.20	0.00	5,901.79	2.20	8.51	1508.345	0.00	728
GFCF	22.29	21.90	38.40	5.40	4.12	0.55	4.83	117.3547	0.00	616
RD	1.39	1.19	3.91	0.20	0.86	0.84	2.87	59.8458	0.00	504
FFEC	76.39	79.85	100.00	12.29	17.92	-1.20	4.06	205.9476	0.00	717
EMPL	5.45	5.30	11.37	0.60	2.59	0.10	2.32	10.67386	0.00	513
INDVA	28.26	28.58	55.85	10.69	6.45	0.27	4.32	57.45635	0.00	682
CPI	20.60	7.50	92.00	2.60	26.08	1.44	3.48	200.6982	0.00	567

Source: Authors’ computations. Notes: For the definition of variables, please see Table 4.

**Table 6:** Correlation matrix.

Variables	GDPC	GDPCG	GGE	GGEI	ESOX	ENOX	ENMVOC	ENH3	GGET	ENVTR	PEC	GIECRE	GFCF	RD	FFEC	EMPL	INDVA	CPI
GDPC	1.00																	
GDPCG	-0.18***	1.00																
GGE	0.56***	-0.03	1.00															
GGEI	-0.08**	0.07*	0.32***	1.00														
ESOX	-0.23***	-0.05	0.00	0.17***	1.00													
ENOX	0.16***	-0.15***	0.08**	0.01	0.79***	1.00												
ENMVOC	0.08**	-0.11***	-0.06	-0.01	0.80***	0.97***	1.00											
ENH3	0.09**	-0.12***	-0.04	-0.07*	0.75***	0.95***	0.96***	1.00										
GGET	0.37***	-0.19***	0.09**	-0.14***	0.63***	0.94***	0.91***	0.91***	1.00									
ENVTR	-0.15***	0.05	-0.06	0.11***	-0.28***	-0.40***	-0.41***	-0.37***	-0.43***	1.00								
PEC	0.21***	-0.17***	0.05	-0.10***	0.72***	0.97***	0.95***	0.94***	0.96***	-0.46***	1.00							
GIECRE	0.13***	-0.14***	-0.21***	-0.25***	0.54***	0.75***	0.81***	0.78***	0.78***	-0.47***	0.81***	1.00						
GFCF	-0.18***	0.31***	0.03	0.01	0.01	-0.11***	-0.08*	-0.10**	-0.17***	-0.08**	-0.12***	-0.02	1.00					
RD	0.69***	-0.23***	0.31***	-0.20***	-0.16***	0.27***	0.24***	0.24***	0.39***	-0.31***	0.39***	0.46***	-0.14***	1.00				
FFEC	0.00	-0.05	0.19***	0.15***	0.10***	0.11***	0.01	0.09**	0.07*	0.38***	0.00	-0.33***	-0.21***	-0.40***	1.00			
EMPL	0.02	0.05	0.04	-0.06	0.36***	0.37***	0.40***	0.40***	0.33***	-0.20***	0.42***	0.26***	0.12***	0.33***	-0.07	1.00		
INDVA	-0.45***	0.22***	-0.15***	0.14***	0.14***	0.07*	0.12***	0.14***	-0.08**	-0.08*	0.07*	0.07*	0.48***	-0.02	-0.08**	0.64***	1.00	
CPI	0.20***	-0.15***	-0.09**	-0.44***	-0.26***	-0.12***	-0.12***	-0.06	-0.00	-0.09**	-0.03	0.12***	-0.31***	0.25***	-0.17***	-0.12***	-0.24***	1.00

Source: Authors’ computations.

*** indicates the statistical significance at 1% levels. For the definition of variables, please see Table 4.

** indicates the statistical significance at 5% levels. For the definition of variables, please see Table 4

* indicates the statistical significance at 10% levels. For the definition of variables, please see Table 4

**Fig 1:**
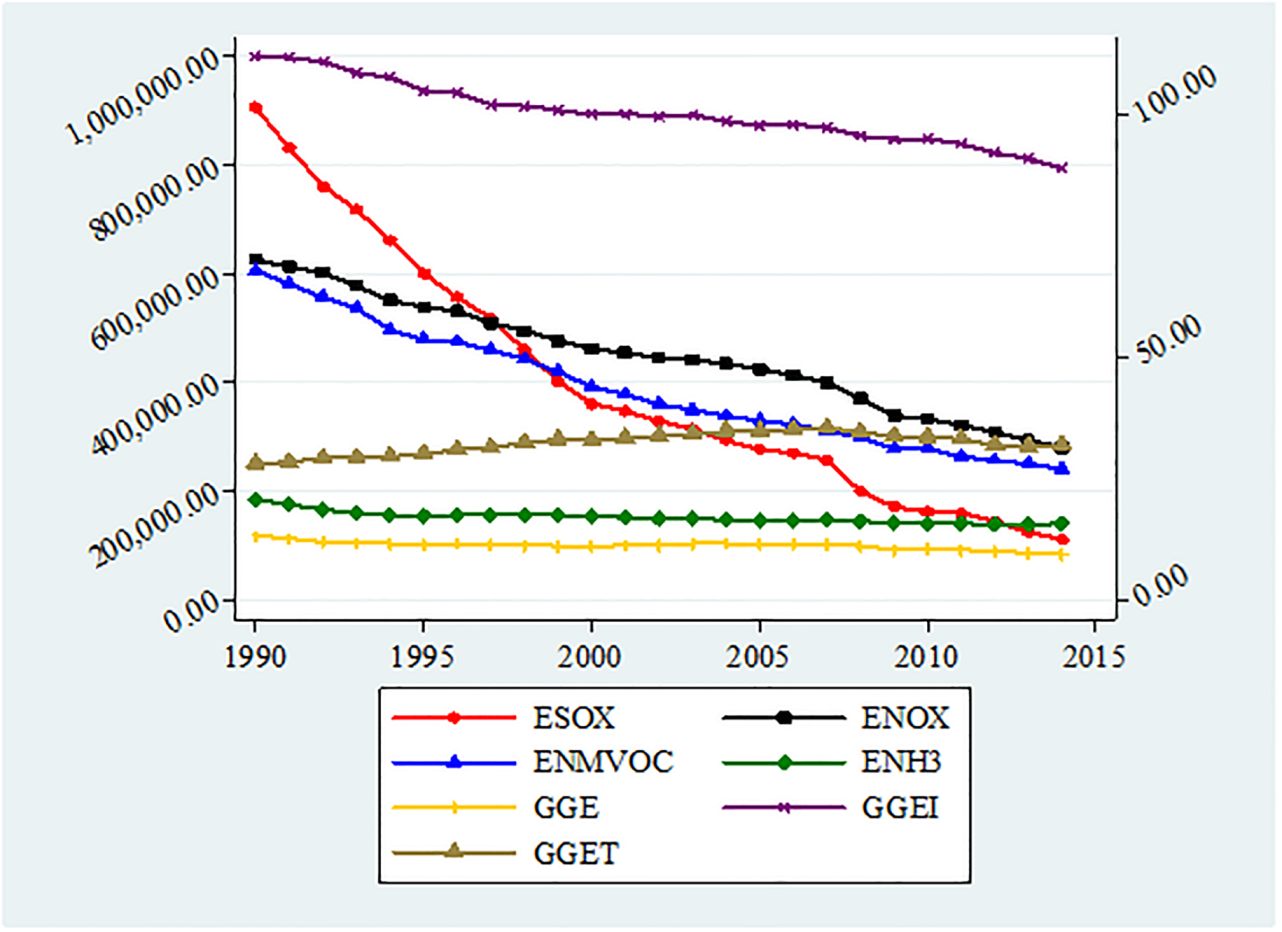
Mean value of pollutant emissions in EU-28. Source: Authors’ own elaboration. Notes: For the definition of variables, please see Table 4.

Fig 1 shows the mean value of pollutant emissions in EU-28 countries. We acknowledge a downward trend of emissions, proving that the European Union is making a considerable effort in order to fulfill the targets related to the second pledge period of the Kyoto Protocol. However, a noteworthy decrease was registered in the case of sulfur oxides determined by shifting from high-sulfur solid and liquid fuels to low sulfur fuels for power and heat fabrication aims within the energy, industry and domestic areas, developments in energy efficiency, and the establishment of flue gas desulfurization equipment in new and current industrial equipment.

The results comprised in Table 7 show the presence of cross-section dependence, similar to the previous findings of [99]. Hence, in order to remove this inconvenience, we considered Driscoll-Kraay standard errors in the fixed-effects models, comparable to [2]. Such standard errors are heteroskedasticity consistent, also being robust to very general forms of cross-sectional and temporal dependence [120].

**Table 7:** Cross-section dependence test results.

Variables	Breusch-Pagan LM	Pesaran scaled LM	Bias-corrected scaled LM	Pesaran CD
GDPC	7607.5***	262.93***	262.37***	86.52***
GDPCG	3024.42***	96.25***	95.69***	50.84***
GGE	3216.89***	103.25***	102.69***	39.3***
GGEI	4899.07***	164.43***	163.85***	55.7***
ESOX	7154.35***	246.45***	245.87***	73.83***
ENOX	4991.48***	167.79***	167.21***	64.54***
ENMVOC	6293.44***	215.14***	214.56***	52.68***
ENH3	3137.43***	100.36***	99.78***	38.47***
GGET	4346.25***	144.32***	143.74***	51.59***
ENVTR	1853.75***	53.67***	52.97***	9.91***
PEC	3271.74***	105.24***	104.68***	16.88***
GIECRE	7254.74***	250.1***	249.54***	84.26***
GFCF	2095.22***	62.45***	61.79***	26.8***
RD	2694.94***	84.27***	83.49***	27.52***
FFEC	4852.64***	162.74***	162.18***	60.86***
EMPL	3208.35***	102.94***	102.2***	35.24***
INDVA	3386.24***	109.41***	108.87***	47.31***
CPI	7165.61***	246.86***	246.2***	84.42***

Source: Authors’ computations.

*** indicates the statistical significance at 1% level. For the definition of variables, please see Table 4.

Further, in line with [121] we perform two categories of stationarity tests, according to their cross-section dependence assumption, respectively the so called first generation that assume cross-section independence (Table 8), as well as the second generation that allow for cross-section dependence between the cross-section units (Table 9). As we can notice, the variables are non-stationary in their levels, but become stationary after taking the first difference. Hence, we conclude that all series are I(1).

**Table 8:** First generation panel unit root test results.

Variables		LLC	IPS	ADF	PP		LLC	Breitung	IPS	ADF	PP
*Levels*							*First differences*				
Intercept	GDPC	-4.62***	0.72	48.89	44.92	Intercept	1.17	-0.42	3.54	37.48	36.02
	GDPCG	-12.89***	-11.77***	239.29***	235.58***		-10.7***	-3.98***	-9.01***	186.48***	198.18***
	GGE	-0.1	0.42	83.89***	83.72***		-1.38*	4.71	0.68	69.23	94.24***
	GGEI	0.38	3.81	55.52	56.8		-3.54***	1.68	-2.54***	91.36***	89.3***
	ESOX	-0.19	6.51	38.27	41.21		-0.74	4.14	1.51	46.33	43.85
	ENOX	2.65	4.81	63.8	69.29		-2.04**	2.5	-0.71	75.94**	79.12**
	ENMVOC	-1.56*	3.93	82.01**	78.04**		-1.31*	3.19	-1.23	85.66***	79.12**
	ENH3	-9.45***	-6.72***	182.32***	166.92***		-6.92***	1.09	-5.85***	146.18***	143.35***
	GGET	-4.17***	-1.17	59.17	62.35		2.34	8.01	3.22	73.94*	56.93
	ENVTR	-1.92**	-1.43*	65.15	68.17		1.08	1.74	0.85	59.89	56.19
	PEC	-5.36***	-3.91***	108.63***	111.86***		-0.08	3.9	1.81	53.28	94.74***
	GIECRE	1.58	5.91	26.76	28.96		-4.13***	-0.79	-2.97***	98.43***	79.76**
	GFCF	-1.59*	-1.91**	80.03**	50.67		-2.9***	-0.76	-3.21***	93.79***	49.73
	RD	-0.43	3.33	38.58	35.42		-0.94	2.4	-0.38	73.19*	74.84**
	FFEC	5.77	8.38	18.82	21.17		1.52	5.46	2.7	57.42	36.09
	EMPL	-1.28	-0.06	68.16	74.63**		-4.78***	-1.55*	-4.02***	105.37***	102.62***
	INDVA	-3.78***	-1.49*	73.02*	82.23**		-11.68***	-0.52	-5.53***	318.2***	48.99
	CPI	4.48	6.1	7.39	7.38		0.28	0.57	3.89	18.46	18.95
Intercept and trend	GDPC	-12.9***	-11.69***	239.01***	242.05***	Intercept and trend	-10.8***	-3.87***	-9.33***	192.57***	228.36***
	GDPCG	-20.83***	-21.1***	445.1***	806.35***		-17.33***	-10.9***	-17.73***	343.66***	1912.96***
	GGE	-15.93***	-15.98***	339.79***	409.63***		-15.49***	-5.97***	-17.5***	342.98***	756.72***
	GGEI	-25.49***	-25.27***	535.4***	740.92***		-22.12***	-10.32***	-23.58***	489.05***	1659.86***
	ESOX	-17.27***	-16.33***	343.84***	359.57***		-17.27***	-10.68***	-16.46***	312.72***	367.78***
	ENOX	-17.37***	-18.04***	373.57***	446.99***		-14.41***	-7.26***	-16.44***	313.00***	1340.09***
	ENMVOC	-16.14***	-15.94***	334.8***	374.03***		-13.28***	-7.35***	-16.05***	306.78***	372.32***
	ENH3	-16.61***	-15.65***	324.43***	421.16***		-15.46***	-6.96***	-13.97***	272.45***	374.08***
	GGET	-12.88***	-12.71***	304.18***	300.72***		-16.07***	-3.43***	-15.56***	353.61***	360.65***
	ENVTR	-12.65***	-12.94***	258.19***	283.55***		-8.7***	-9.47***	-10.63***	204.28***	266.51***
	PEC	-18.65***	-18.21***	385.09***	490.16***		-19.08***	-8.36***	-20.91***	480.65***	857.53***
	GIECRE	-21.39***	-21.43***	441.63***	492.82***		-18.23***	-13.24***	-18.5***	371.38***	1087.21***
	GFCF	-11.81***	-11.8***	239.18***	260.53***		-9.94***	-5.82***	-8.9***	177.47***	218.2***
	RD	-8.38***	-9.37***	195.89***	508.07***		-8.82***	-5.01***	-8.45***	174.26***	233.05***
	FFEC	-16.95***	-17.32***	371.29***	412.5***		-14.88***	-6.72***	-16.24***	319.42***	677.16***
	EMPL	-17.66***	-15.56***	310.33***	427.82***		-14.99***	-7.68***	-10.78***	221.95***	300.8***
	INDVA	-19.7***	-17.65***	362.45***	360.33***		-15.69***	-9.42***	-14.15***	270.04***	349.4***
	CPI	-20.25***	-15.07***	299.1***	304.61***		-18.74***	-6.08***	-12.36***	230.66***	243.6***

Source: Authors’ computations. Notes: lag lengths are determined via Schwarz Info Criterion. LLC reveals Levin, Lin and Chu t* stat. IPS reveals Im, Pesaran and Shin W-stat. ADF reveals Augmented Dickey-Fuller Fisher Chi-square. PP reveals Phillips–Perron Fisher Chi-square. LLC assumes common unit root process. IPS, ADF, and PP assumes individual unit root process. Probabilities for ADF and PP are computed using an asymptotic Chi-square distribution. Probabilities for the LLC, Breitung, and IPS tests are computed assuming asymptotic normality. For the definition of variables, please see Table 4.

*** indicates the statistical significance at 1% levels.

** indicates the statistical significance at 5% levels.

* indicates the statistical significance at 10% levels.

**Table 9:** Second generation panel unit root investigation—Pesaran’s CADF test.

Deterministics chosen	Variables	Levels							First differences					
		t-bar	cv10	cv5	cv1	Z[t-bar]	P-value		t-bar	cv10	cv5	cv1	Z[t-bar]	P-value
Constant	GDPC	-1.39	-2.07	-2.15	-2.30	1.95	0.97	Constant	-2.65	-2.07	-2.15	-2.30	-4.82	0.00
	GDPCG					-5.78	0.00						-9.50	0.00
	GGE					-0.12	0.45						-7.68	0.00
	GGEI	-2.03	-2.07	-2.15	-2.30	-1.51	0.07		-3.90	-2.07	-2.15	-2.30	-11.60	0.00
	ESOX	-1.66	-2.07	-2.15	-2.30	0.47	0.68		-2.95	-2.07	-2.15	-2.30	-6.48	0.00
	ENOX	-2.29	-2.07	-2.15	-2.30	-2.90	0.00		-3.71	-2.07	-2.15	-2.30	-10.60	0.00
	ENMVOC	-1.98	-2.07	-2.15	-2.30	-1.23	0.11		-2.86	-2.07	-2.15	-2.30	-6.01	0.00
	ENH3	-2.33	-2.07	-2.15	-2.30	-3.15	0.00		-3.56	-2.07	-2.15	-2.30	-9.75	0.00
	GGET	-2.15	-2.07	-2.15	-2.30	-2.15	0.02		-3.24	-2.07	-2.15	-2.30	-8.06	0.00
	ENVTR					-1.10	0.14						-4.59	0.00
	PEC	-2.08	-2.07	-2.15	-2.30	-1.81	0.04		-3.66	-2.07	-2.15	-2.30	-10.30	0.00
	GIECRE	-2.24	-2.07	-2.15	-2.30	-2.62	0.00		-3.79	-2.07	-2.15	-2.30	-11.03	0.00
	GFCF	-1.80	-2.07	-2.15	-2.30	-0.25	0.40		-2.89	-2.07	-2.15	-2.30	-6.14	0.00
	RD	-1.92	-2.07	-2.15	-2.32	-0.94	0.17						-2.91	0.00
	FFEC					0.40	0.65						-8.91	0.00
	EMPL					-2.69	0.00						-5.50	0.00
	INDVA					-0.34	0.37						-6.85	0.00
	CPI					-6.46	0.00						-9.44	0.00
Constant & Trend	GDPC	-2.24	-2.58	-2.66	-2.81	0.41	0.66	Constant & Trend	-2.69	-2.58	-2.66	-2.81	-2.16	0.02
	GDPCG					-2.69	0.004						-6.41	0.00
	GGE					2.29	0.989						-9.14	0.00
	GGEI	-2.37	-2.58	-2.66	-2.81	-0.33	0.37		-4.22	-2.58	-2.66	-2.81	-10.87	0.00
	ESOX	-1.96	-2.58	-2.66	-2.81	2.00	0.977		-3.19	-2.58	-2.66	-2.81	-5.01	0.00
	ENOX	-2.71	-2.58	-2.66	-2.81	-2.29	0.01		-3.91	-2.58	-2.66	-2.81	-9.09	0.00
	ENMVOC	-2.02	-2.58	-2.66	-2.81	1.64	0.949		-3.23	-2.58	-2.66	-2.81	-5.24	0.00
	ENH3	-2.35	-2.58	-2.66	-2.81	-0.24	0.404		-3.75	-2.58	-2.66	-2.81	-8.17	0.00
	GGET	-2.53	-2.58	-2.66	-2.81	-1.25	0.105		-3.34	-2.58	-2.66	-2.81	-5.85	0.00
	ENVTR					2.50	0.99						-3.41	0.00
	PEC	-2.18	-2.58	-2.66	-2.81	0.76	0.78		-4.33	-2.58	-2.66	-2.81	-11.50	0.00
	GIECRE	-2.61	-2.58	-2.66	-2.81	-1.69	0.05		-4.06	-2.58	-2.66	-2.81	-9.95	0.00
	GFCF	-2.50	-2.58	-2.66	-2.81	-1.05	0.15		-3.02	-2.58	-2.66	-2.81	-4.01	0.00
	RD	-2.17	-2.58	-2.67	-2.83	0.61	0.73						-0.37	0.36
	FFEC					1.62	0.947						-7.89	0.00
	EMPL					-0.75	0.23						-3.51	0.00
	INDVA					1.35	0.911						-5.16	0.00
	CPI					-4.75	0.00						-6.31	0.00

Source: Authors’ computations. Notes: For the definition of variables, please see Table 4.

### Panel regression analysis

Table 10 provides the results of pooled OLS regressions with Driscoll-Kraay standard errors. According to F statistic, all the estimated models are statistically highly significant, and the values related to R-squared reveal that it could explain between 32% and 98% of the variability in pollutant emissions. The coefficients related to GDP per capita and squared GDP per capita are statistically significant in all the estimated models, except the models 4, 6 and 7. Nevertheless, the EKC hypothesis is confirmed in case of ESOX and ENMVOC. Furthermore, the output of fixed-effects regressions with Driscoll-Kraay standard errors is showed in Table 11. The estimated regressions appear to fit the data rather well since they can explain almost 42% and 87% of the pollutant emissions variation. An inverted U-shaped curve emerges in case of all selected harming emanations, except ESOX and GGET. With regard to the environmental tax revenues, we acknowledge that the expectation concerning ecological damage reduction is not supported since almost all the estimated models show a positive influence of such taxes on pollutant emissions. While primary energy consumption drives pollution, we notice, with some exceptions, that renewable energies consumption reduces pollutant emissions, as previously found by [56]. In terms of research and development expenditure, like [51], our results reinforce the beneficial effect of innovation on environmental pollution. As regards the variables employed to control for the scale effect, technique effect and political condition, we ascertain mixed evidence.

**Table 10:** Pooled OLS regressions with Driscoll-Kraay standard errors.

Independent variables	Dependent variables						
	GGE (1)	GGEI (2)	ESOX (3)	ENOX (4)	ENMVOC (5)	ENH3 (6)	GGET (7)
GDPC	-2.05***	-0.53***	4.66***	-0.43	1.67***	-1.05	0.58*
	(-4.95)	(-4.67)	(6.03)	(-1.02)	(3.13)	(-1.74)	(1.84)
GDPCSQ	0.13***	0.03***	-0.27***	0.03	-0.08***	0.06*	-0.01
	(5.67)	(4.77)	(-6.56)	(1.28)	(-3.04)	(1.93)	(-0.45)
ENVTR	0.02**	0.00	0.10***	0.03**	0.03**	0.05***	-0.01**
	(2.99)	(1.06)	(4.23)	(2.21)	(2.33)	(3.77)	(-2.55)
PEC	0.13***	0.01	1.48***	0.94***	0.83***	0.89***	0.80***
	(10.17)	(1.35)	(23.32)	(47.65)	(21.28)	(33.03)	(41.61)
GIECRE	-0.14***	-0.01*	-0.35***	0.04***	0.19***	0.12***	0.13***
	(-6.14)	(-1.81)	(-6.50)	(3.58)	(9.02)	(5.74)	(15.59)
GFCF	0.01**	-0.00	0.02	0.00	-0.02***	-0.01*	-0.01*
	(2.93)	(-0.09)	(1.27)	(0.42)	(-3.17)	(-1.82)	(-1.98)
RD	-0.06	0.01	-0.34*	-0.17***	-0.37***	-0.35***	-0.27***
	(-1.63)	(1.08)	(-2.05)	(-3.67)	(-7.99)	(-5.73)	(-6.30)
FFEC	-0.00***	0.00	-0.01*	0.00**	-0.00	0.00	0.00***
	(-5.05)	(0.88)	(-1.86)	(2.75)	(-1.06)	(1.14)	(3.00)
EMPL	-0.01**	-0.00	-0.06*	-0.02**	0.01	-0.01	0.01***
	(-2.53)	(-1.14)	(-1.92)	(-2.99)	(0.62)	(-0.99)	(3.22)
INDVA	0.02***	0.00	0.03***	0.00	0.02***	0.04***	-0.01***
	(8.98)	(0.64)	(6.54)	(0.83)	(3.07)	(9.92)	(-4.10)
CPI	-0.00	-0.00***	-0.00**	-0.00***	-0.00***	0.00	-0.00***
	(-1.42)	(-4.34)	(-2.71)	(-3.89)	(-3.76)	(1.56)	(-6.48)
_cons	10.16***	7.26***	-10.98**	10.28***	-0.79	10.77***	-5.47***
	(5.57)	(12.58)	(-2.89)	(4.67)	(-0.31)	(3.75)	(-3.45)
F statistic	4274.60***	71.77***	173364.36***	154719.35***	67864.83***	117622.81***	219042.44***
R-sq	0.63	0.32	0.83	0.97	0.96	0.92	0.98
Obs	470	470	470	470	470	470	470
N Countries	28	28	28	28	28	28	28

Source: Authors’ computations. Numbers in the parentheses represent t-stat values. For the definition of variables, please see Table 4.

*** indicates the statistical significance at 1% levels.

** indicates the statistical significance at 5% levels.

* indicates the statistical significance at 10% levels.

**Table 11:** Fixed-effects regressions with Driscoll-Kraay standard errors.

Independent variables	Dependent variables						
	GGE (1)	GGEI (2)	ESOX (3)	ENOX (4)	ENMVOC (5)	ENH3 (6)	GGET (7)
GDPC	2.68***	0.84**	-0.99	1.62***	5.15***	1.42**	0.84***
	(6.70)	(2.76)	(-0.74)	(3.49)	(8.78)	(2.57)	(5.33)
GDPCSQ	-0.14***	-0.05***	-0.03	-0.10***	-0.29***	-0.08**	-0.01
	(-6.43)	(-3.01)	(-0.44)	(-4.12)	(-8.54)	(-2.63)	(-1.53)
ENVTR	0.00	0.00	0.01	0.01	-0.01	0.02***	0.00
	(0.67)	(0.46)	(0.42)	(1.59)	(-1.35)	(4.32)	(0.01)
PEC	0.69***	0.02	0.85***	0.64***	0.14	0.31***	0.39***
	(19.67)	(0.53)	(7.10)	(7.11)	(1.54)	(5.41)	(5.64)
GIECRE	-0.02***	0.02***	-0.13*	-0.04	0.04**	-0.02	0.02***
	(-3.02)	(3.00)	(-1.99)	(-1.76)	(2.42)	(-1.48)	(4.56)
GFCF	-0.00**	-0.00**	0.02***	0.00	0.00	0.00*	0.00**
	(-2.27)	(-2.79)	(3.42)	(1.66)	(1.20)	(2.08)	(2.62)
RD	0.04**	0.02**	-0.23***	0.01	-0.13***	0.03	0.03
	(2.98)	(2.20)	(-3.73)	(0.53)	(-3.92)	(1.27)	(1.61)
FFEC	0.01***	0.01***	0.04***	0.02***	0.01***	0.01***	0.00*
	(17.95)	(18.02)	(8.90)	(19.87)	(4.58)	(8.02)	(1.96)
EMPL	0.01***	-0.00	0.05	0.05***	0.07***	0.02***	0.02***
	(3.86)	(-0.91)	(1.15)	(5.51)	(4.63)	(3.74)	(4.24)
INDVA	0.01***	0.01***	0.08***	0.01***	0.03***	0.01*	-0.01***
	(7.54)	(6.39)	(6.28)	(5.88)	(4.06)	(1.85)	(-3.74)
CPI	-0.00	-0.00*	0.00**	-0.00*	-0.00**	0.00***	-0.00***
	(-0.72)	(-1.99)	(2.23)	(-1.87)	(-2.47)	(3.03)	(-6.87)
_cons	-14.22***	-0.29	17.49***	2.06	-13.04***	3.09	-6.11***
	(-7.63)	(-0.19)	(3.40)	(0.95)	(-4.94)	(1.21)	(-7.29)
F statistic	7045.93***	1520.21***	1487.69***	4101.01***	325.65***	386.02***	1287.52***
within R-sq	0.87	0.81	0.76	0.82	0.72	0.42	0.71
Obs	470	470	470	470	470	470	470
N Countries	28	28	28	28	28	28	28

Source: Authors’ computations. Numbers in the parentheses represent t-stat values. For the definition of variables, please see Table 4.

*** indicates the statistical significance at 1% levels.

** indicates the statistical significance at 5% levels.

* indicates the statistical significance at 10% levels.

**Fig 2:**
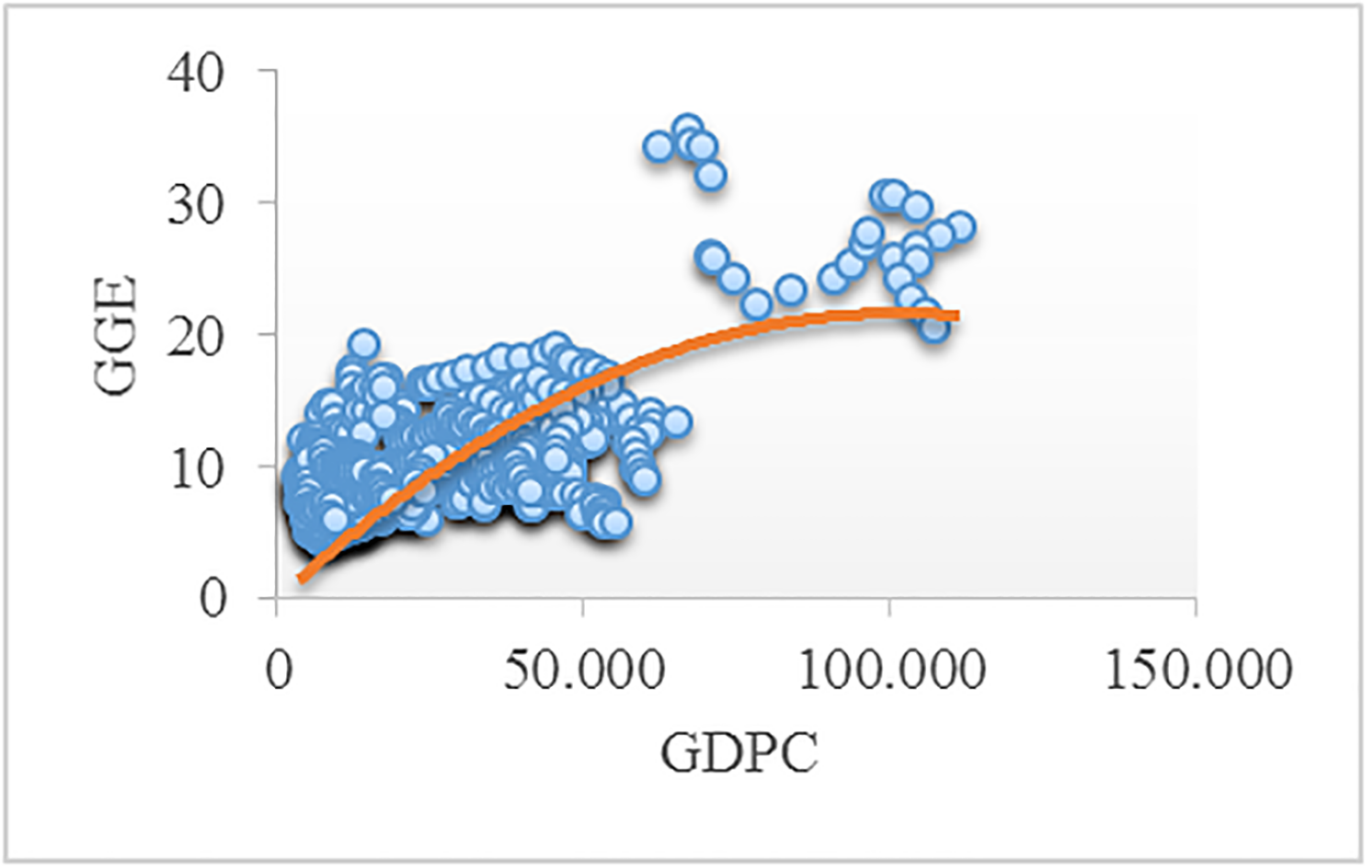
Plotted graphs between GDP per capita and greenhouse gas emissions in EU-28. Source: Authors’ own elaboration.

**Fig 3:**
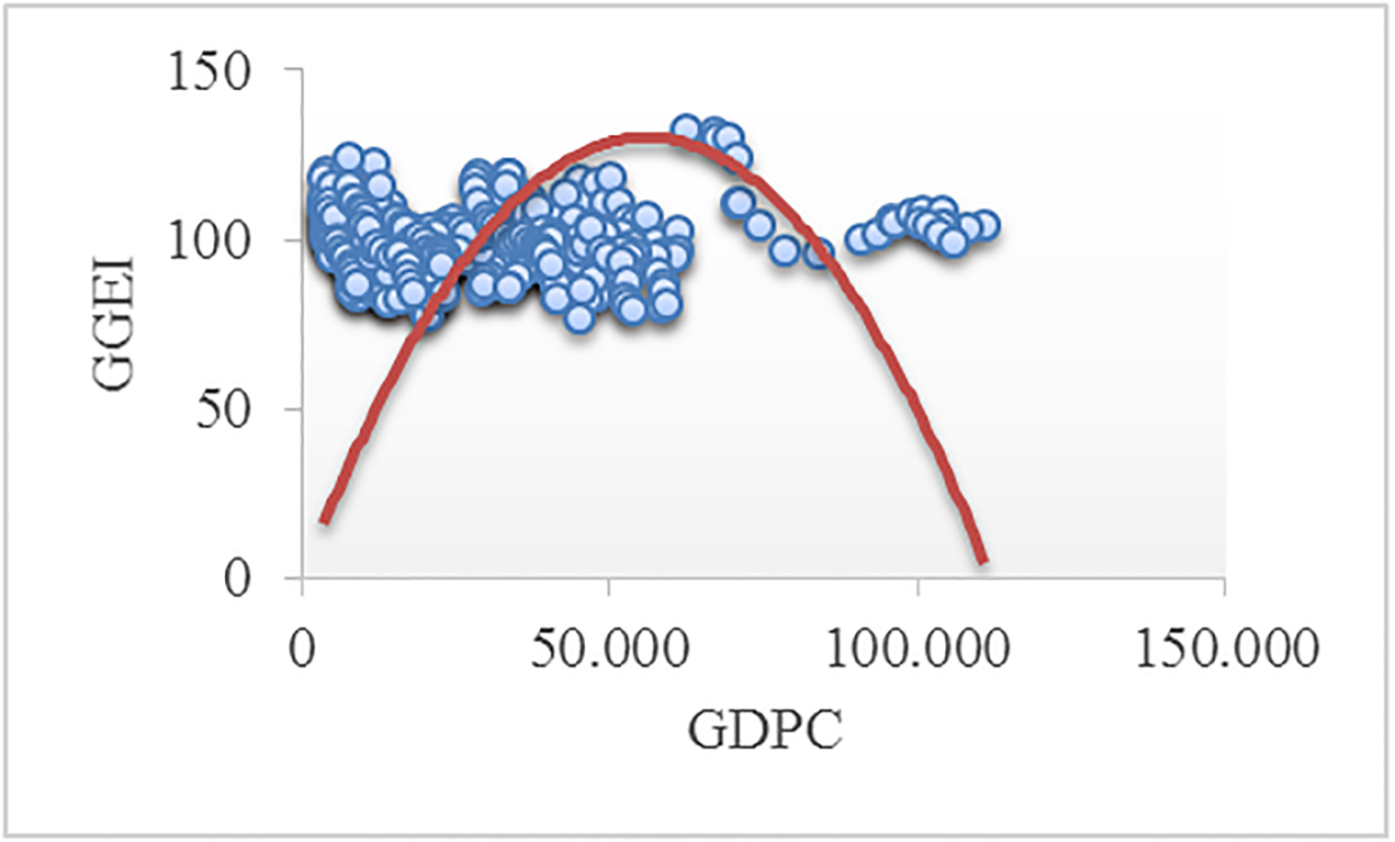
Plotted graphs between GDP per capita and greenhouse gas emissions intensity of energy consumption in EU-28. Source: Authors’ own elaboration.

**Fig 4:**
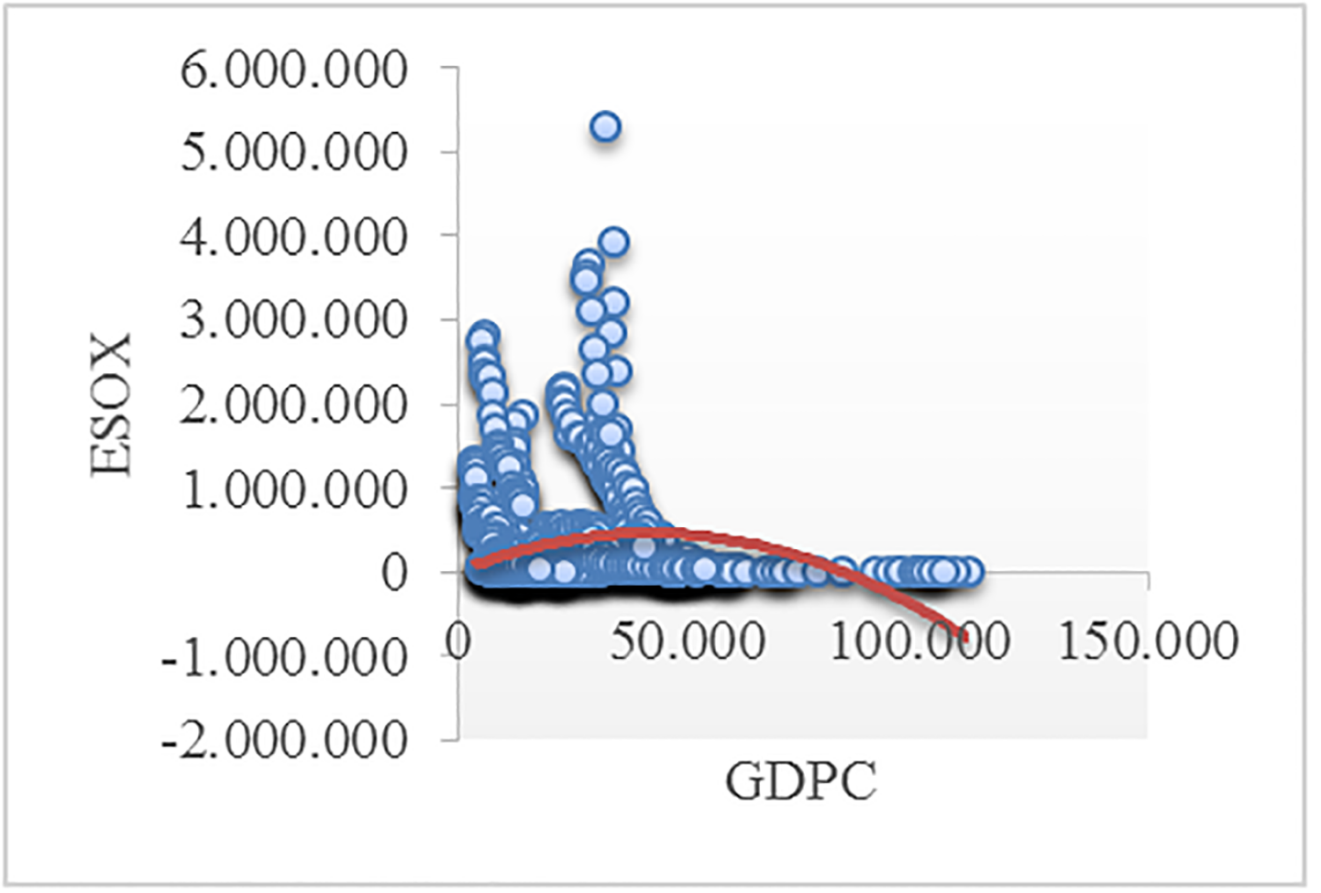
Plotted graphs between GDP per capita and emissions of sulfur oxides in EU-28. Source: Authors’ own elaboration.

**Fig 5:**
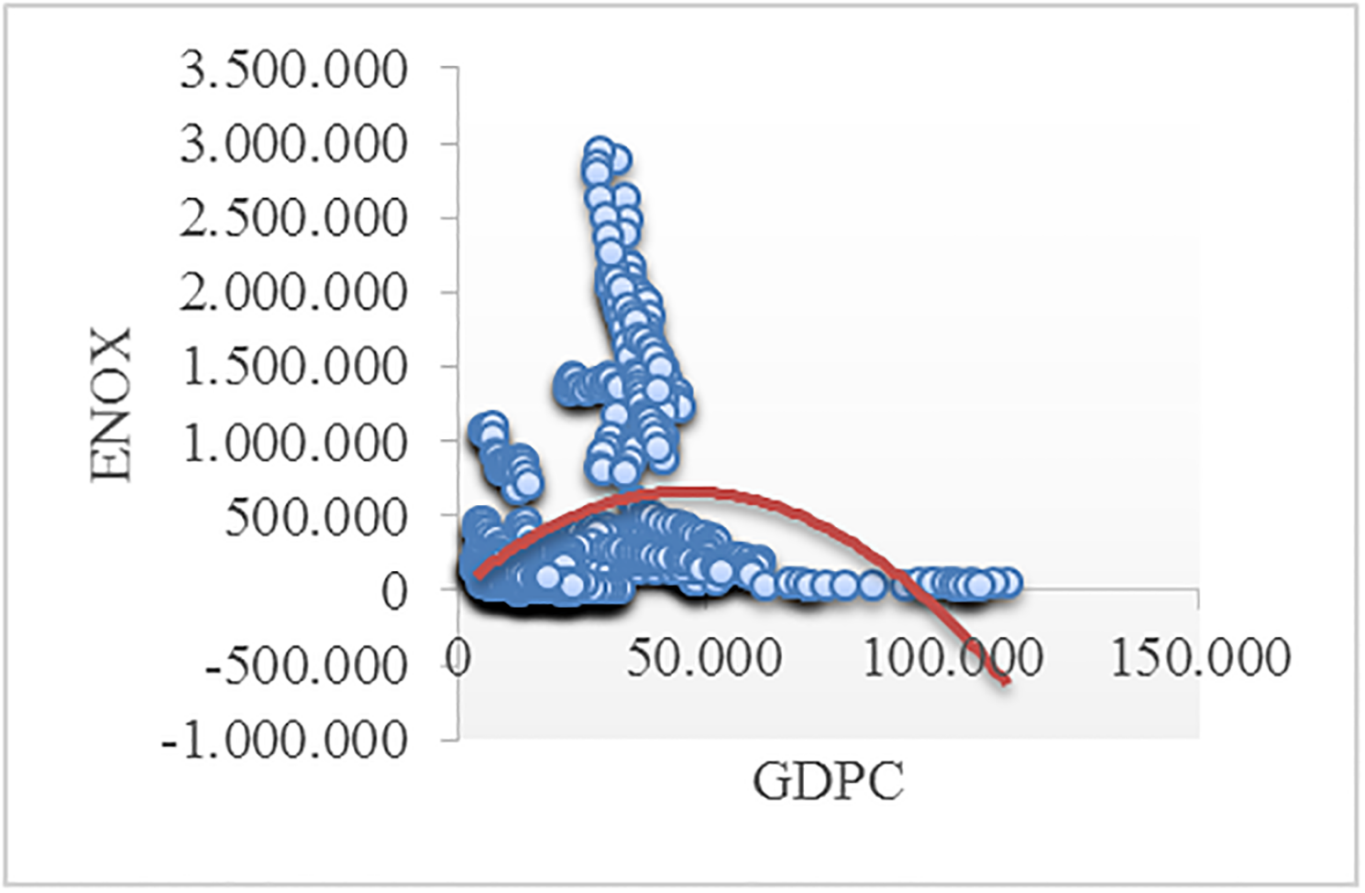
Plotted graphs between GDP per capita and emissions of nitrogen oxides in EU-28. Source: Authors’ own elaboration.

**Fig 6:**
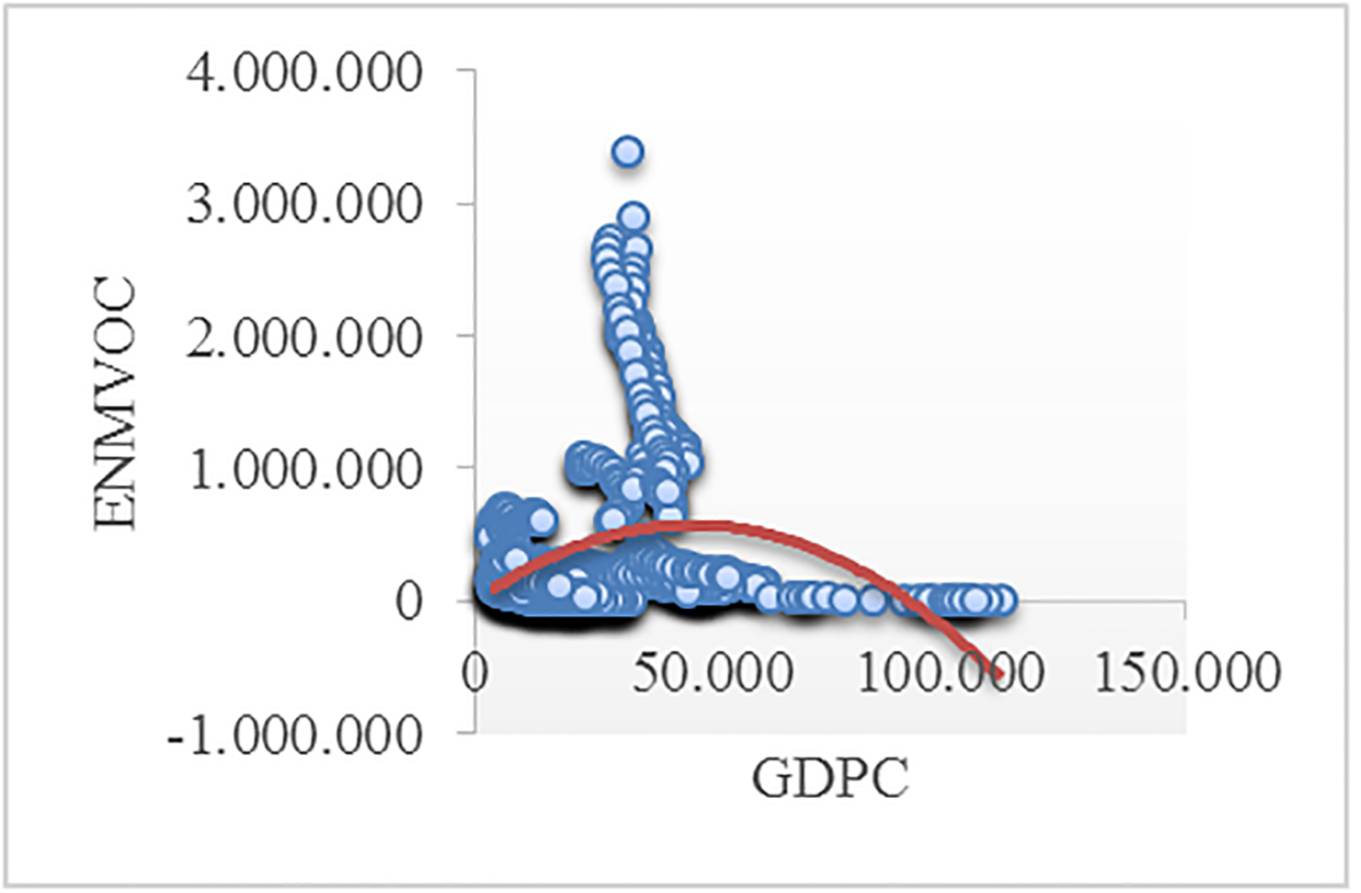
Plotted graphs between GDP per capita and emissions of non-methane volatile organic compounds in EU-28. Source: Authors’ own elaboration.

**Fig 7:**
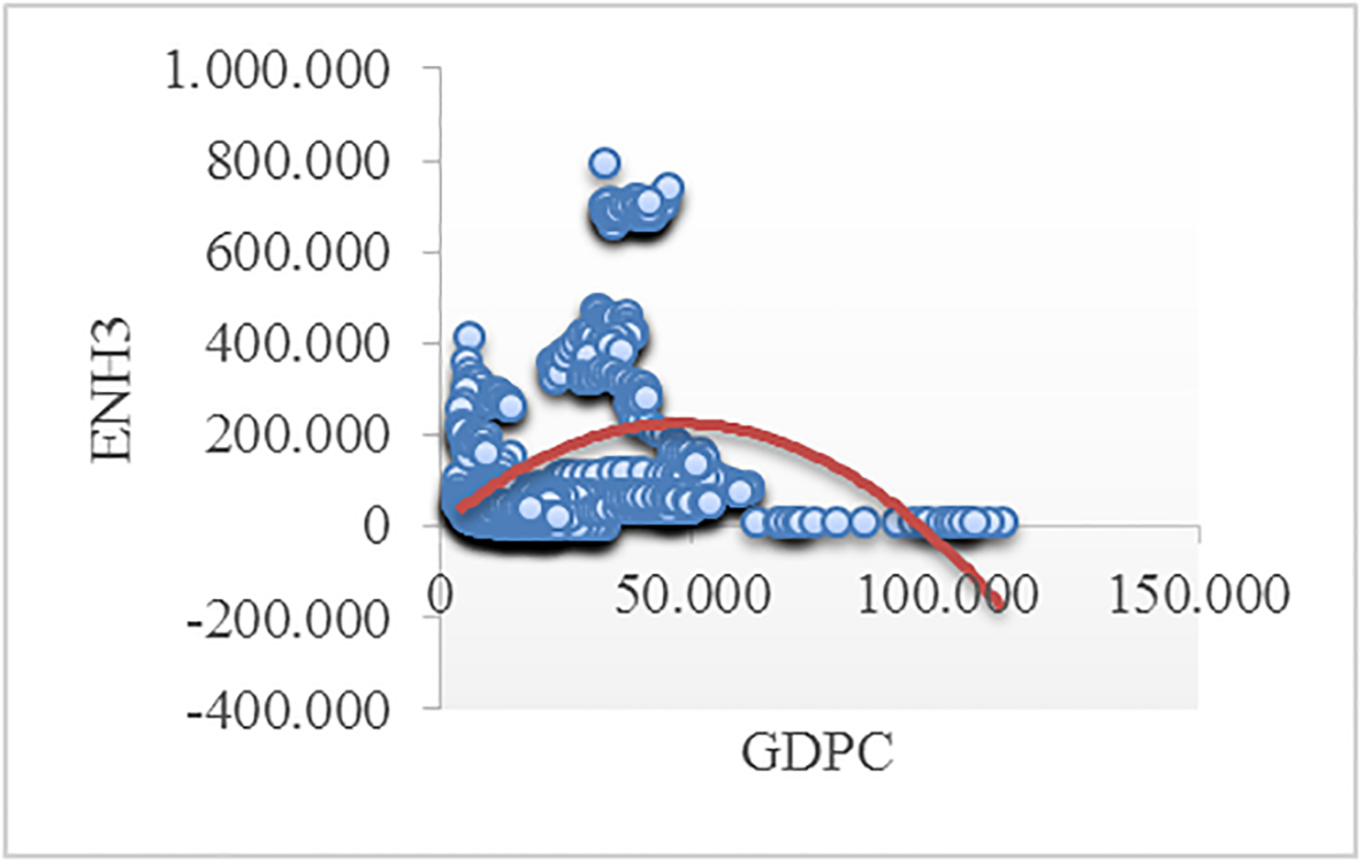
Plotted graphs between GDP per capita and emissions of ammonia in EU-28. Source: Authors’ own elaboration.

**Fig 8:**
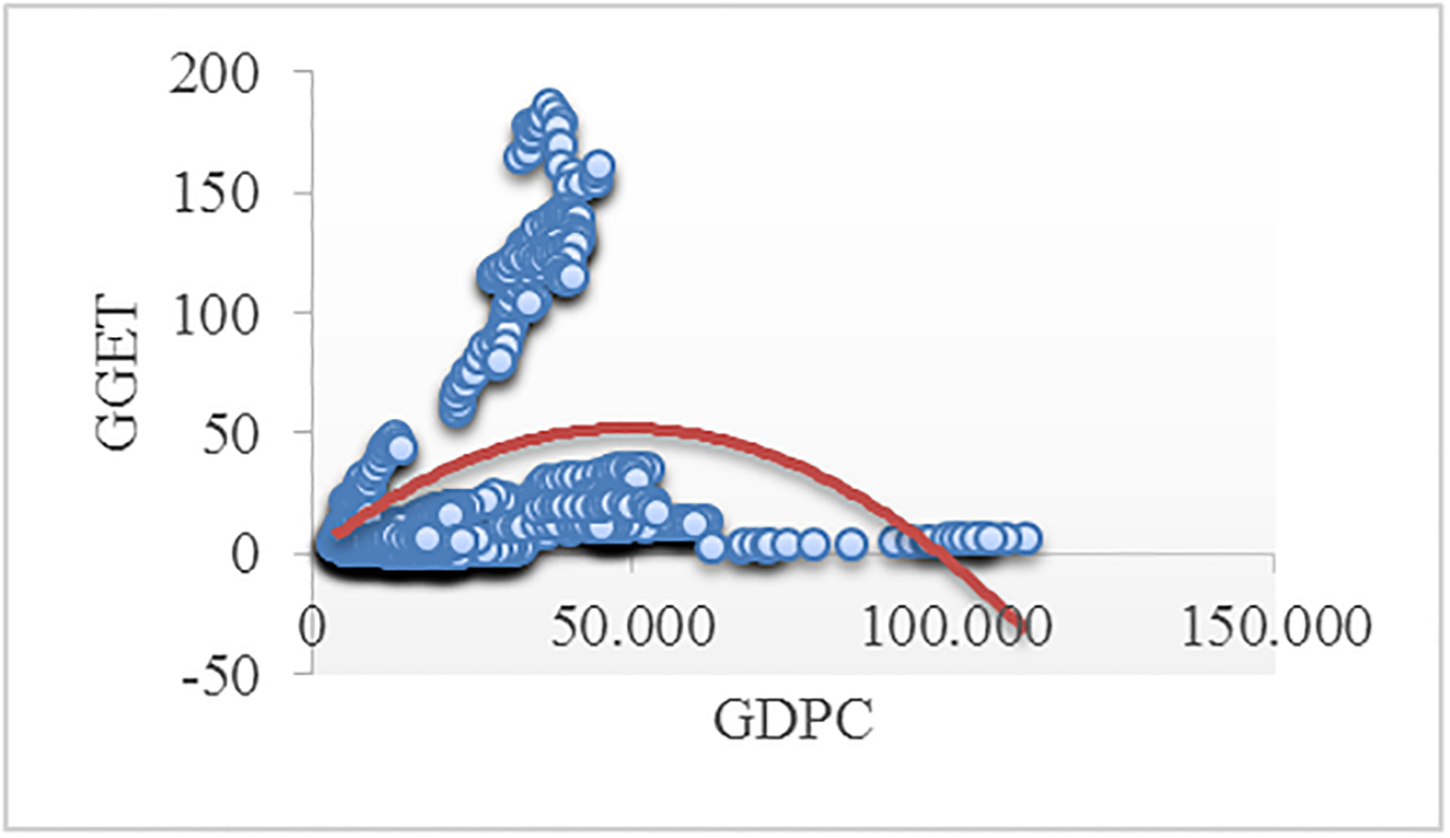
Plotted graphs between GDP per capita and greenhouse gas emissions from transport in EU-28. Source: Authors’ own elaboration.

Figs 2–8 reveals the plotted graphs between GDP per capita and pollutant emissions.

The EKC hypothesis appears to be sustained since the inversed U-shaped curve tends to fit properly at least for greenhouse gas emissions. In addition, Table 12 exhibits the estimated turning points. We notice that the values of the established turning points are not in line with previous studies [38, 39] that employed GDP per capita (in 2010 U.S. $). However, alike [37, 63, 65, 97, 122], we notice values which lie outside the sample, namely for GGE (pooled OLS estimations) and ENOX (fixed-effects estimations). Moreover, [17] underlined a higher likelihood of identifying turning points outside the sample in case of developing countries than developed countries.

**Table 12:** Estimated turning points from current study (in 2010 U.S. $).

Panel regression analysis	GGE	GGEI	ESOX	ENOX	ENMVOC	ENH3	GGET
Pooled OLS regressions	3,570 (outside the sample)	20,895	6,097	-	43,393	-	-
Fixed-effects regressions	18,041.52	5,691.36	-	2,885.07 (outside the sample)	8,115.80	7,835.30	-

Source: Authors’ own elaboration. Notes: For the definition of variables, please see Table 4.

### Co-integration and causality investigation

The outcome of Pedroni panel co-integration test [115, 116] is presented in Table 13. According to [88, 89, 104], the statistics based on the dimension approach pools the autoregressive coefficients across different states for the unit root tests on the estimated residuals considering common time factors and heterogeneity across countries. To examine the null hypothesis of no co-integration, *ρ*_*i*_ = 1, the following unit root test towards the residuals [88, 89, 104] is performed:

$$\varepsilon_{it}=\rho_{i}\varepsilon_{it-1}+w_{it}$$ (4)

**Table 13:** Pedroni (Engle Granger based) test results.

Panel A: Within-dimension				
Panel co-integration test	Individual intercept		Individual intercept and individual trend	
	Statistic	Weighted Statistic	Statistic	Weighted Statistic
Panel v-Statistic	1.87**	-1.25	-0.90	-4.31
Panel rho-Statistic	0.71	-0.01	3.02	2.07
Panel PP-Statistic	-3.22***	-8.16***	-4.52***	-9.12***
Panel ADF-Statistic	-7.45***	-9.91***	-7.87***	-9.62***
Panel B: Between-dimension				
Panel co-integration test	Statistic		Statistic	
Group rho-Statistic	2.51		4.37	
Group PP-Statistic	-10.74***		-11.59***	
Group ADF-Statistic	-10.34***		-9.46***	

Source: Authors’ computations. Schwarz Info Criterion was selected for lag length.

*** indicates the statistical significance at 1% levels

** indicates the statistical significance at 5% levels

* indicates the statistical significance at 10% levels

As shown in panel A, panel PP and panel ADF statistics strongly reject the null hypothesis of no co-integration. Additionally, the statistics based on the between-dimension are based on the mean values of the individual autoregressive coefficients related to the unit root tests of the residuals for each member state. The results from panel B reinforce that panel PP and panel ADF statistics reject the null hypothesis of no co-integration at the significance level of 1%.

The second test of panel co-integration employed within current examination is the Kao test (the outcome is reported in Table 14). [117] suggests an ADF panel co-integration test where the vectors of co-integration are homogeneous. However, according to [123], this test is based on a panel version of the ADF test on the residual (*ε*_*it*_):

$$\varepsilon_{it}=\rho_{i}\varepsilon_{it-1}+\sum_{j=1}^{k}\gamma_{j}\Delta \varepsilon_{i,t-j}+w_{it}$$ (5)

**Table 14:** Kao (Engle Granger based) test results.

ADF (t-Statistic)	Residual variance	HAC variance
-2.94***	11.52	3.76

Source: Authors’ computations. Schwarz Info Criterion was selected for lag length.

*** indicates the statistical significance at 1% levels.

** indicates the statistical significance at 5% levels.

* indicates the statistical significance at 10% levels.

Further, the following ADF statistic is produced:

$$\text{ADF}=\frac{\tau_{ADF}+\;\sqrt{6N}{\hat{\sigma }}_{v}/\left(2{\hat{\sigma }}_{0v}\right)}{\sqrt{{\hat{\sigma }}_{0v}^{2}/\left(2{\hat{\sigma }}_{v}^{2}\right)\;+3{\hat{\sigma }}_{v}^{2}/\left(10{\hat{\sigma }}_{0v}^{2}\right)}}$$ (6)

where ${\hat{\sigma }}_{v}^{2}$ depicts the estimated variance, ${\hat{\sigma }}_{0v}^{2}$ describes the estimated long-run variance of the error term which follows the standard normal distribution, and *τ*_*ADF*_ reveals the ADF statistic for Eq (5). Thus, since the probability of ADF is 0.0274, the results provide support for the hypothesis of co-integration among all variables.

The third co-integration approach used is depicted by Fisher-type panel co-integration test [118] showed in Table 15. The Johansen Fisher panel co-integration test aggregates the p-values of individual Johansen maximum eigen-value and trace statistics [124]; this test also rejects the null hypothesis of no co-integration.

**Table 15:** Fisher (combined Johansen) test results.

Hypothesized No. of CE(s)	Fisher Stat. (from trace test)	Fisher Stat. (from max-eigen test)
None	380.2***	299.7***
At most 1	160.3***	111.5***
At most 2	95.9***	76.11**
At most 3	96.44***	96.44***

Source: Authors’ computations. Schwarz Info Criterion was selected for lag length. Probabilities are computed using asymptotic Chi-square distribution.

*** indicates the statistical significance at 1% levels.

** indicates the statistical significance at 5% levels.

* indicates the statistical significance at 10% levels.

Further, the Westerlund test [119] is employed (the outcome is showed in Table 16). Contrary to [121], the null hypothesis of no cointegration between greenhouse gas emissions, economic growth, primary energy consumption, and environmental tax revenues is rejected. Therefore, when the cross-dependence is considered in the panel, there exists a long-run equilibrium relationship between the variables.

**Table 16:** The output of Westerlund panel cointegration test.

Statistic	Value	Z-value	P-value
Gt	-2.80	-5.64	0.00
Ga	-5.44	2.01	0.98
Pt	-11.14	-3.18	0.00
Pa	-5.49	-1.02	0.15

Source: Authors’ computations. Note: Gt and Ga reveal the group mean statistics that examine the null of no cointegration for the whole panel against the alternative of cointegration. Pt and Pa are the panel statistics that test the null of no cointegration against the alternative of cointegration for the panel as a whole. Bartlett-Kernel window width set according to 4(T/100)^2/9^ ≈ 3.

Onward, since the variables are co-integrated, a panel vector error correction model is estimated in order to perform Granger-causality tests presented in Table 17. The panel error correction model, Eqs (3a)–(3d), allows two sources of causality, namely short-run causality via the lagged difference terms, as well as long-run causality by mean values of the error correction terms. With respect to Eq (3a), environmental tax revenues show a statistically significant influence on the short-run on GDP per capita growth. Concerning Eq (3b), there ensues a unidirectional causality running from economic growth to greenhouse gas emissions as in [96, 102]. In addition, primary energy consumption has a statistically significant effect on GHG emissions. In Eq (3c), we noticed the statistically significant influence of GHG emissions and primary energy consumption on environmental tax revenues. Eq (3d) shows that GHG emissions and environmental tax revenues have a statistically significant impact on primary energy consumption. However, in as much as there is no causal link between GDPCG and PEC, the neo-classical view is confirmed, respectively the neutrality assumption. Moreover, the error correction term is statistically significant, but reveals a relatively slow speed of adjustment towards equilibrium.

**Table 17:** Granger causality based on panel vector error correction model.

Excluded	Short-run causality				Long-run causality
	Dependent variable				
	ΔGDPCG	ΔGGE	ΔENVTR	ΔPEC	ECT
ΔGDPCG	-	10.94***	2.17	3.04	-0.40***
ΔGGE	1.87	-	7.59**	13.18***	0.003***
ΔENVTR	6.74**	4.41	-	17.11***	-0.02**
ΔPEC	2.35	8.97**	8.12**	-	0.002**

Source: Authors’ computations. For the definition of variables, please see Table 4.

*** indicates the statistical significance at 1% levels.

** indicates the statistical significance at 5% levels.

* indicates the statistical significance at 10% levels.

## Conclusions

The objective of the present study was to firstly examine the EKC hypothesis and subsequently the causal relationships between greenhouse gas emissions, economic growth, primary energy consumption, and environmental tax revenues, for a panel consisting of the EU-28 countries over the period 1990–2014. Since we noticed cross-sectional dependence in each of the variables in the panel, we employed Driscoll-Kraay standard errors. The results of pooled OLS regressions confirmed the EKC hypothesis for emissions of sulfur oxides and emissions of non-methane volatile organic compounds, whereas the outcome of fixed-effects estimations validated furthermore the EKC approach for greenhouse gas emissions, greenhouse gas emissions intensity of energy consumption, emissions of nitrogen oxides, emissions of non-methane volatile organic compounds and emissions of ammonia. Additionally, the estimation of a panel vector error correction model shows the presence of a short-run unidirectional causality from GDP per capita growth to greenhouse gas emissions, as well as a bidirectional causal link between primary energy consumption and greenhouse gas emissions. Besides, the neo-classical view was endorsed, respectively the neutrality hypothesis.

The main policy implication deriving from our research can be formulated as follows: EU-28 states should promote the use of renewable energies that are constantly replenished and which will never end. Accordingly, the use of renewable energy will contribute to the decrease of GHGs emissions, while also reducing the reliance on fossil fuel markets. Besides, EU-28 may benefit from enhanced employment opportunities due to jobs occurrence in new cleaner technologies. Finally, as future research endeavors, our aim is to extend the empirical analysis in order to test the EKC hypothesis utilizing a composite index of environmental performance.
